# Association Between High-Density Lipoprotein Cholesterol Levels and Sarcopenia in Elderly Koreans

**DOI:** 10.3390/jcm15010183

**Published:** 2025-12-26

**Authors:** Jun-Young Huh, Junghwan Cho, Hye Rang Bak

**Affiliations:** 1 Department of Family Medicine, Samsung Changwon Hospital, Sungkyunkwan University School of Medicine, Changwon 51353, Republic of Korea; paradisor@naver.com; 2 Division of Endocrinology & Metabolism, Department of Internal Medicine, Samsung Changwon Hospital, Sungkyunkwan University School of Medicine, Changwon 51353, Republic of Korea

**Keywords:** aging, cholesterol, HDL, lipoproteins, muscles, sarcopenia

## Abstract

**Background/Objectives**: High-density lipoprotein cholesterol (HDL-C) regulates muscle energy metabolism and function, enhancing glucose uptake and promoting glycogen synthesis. However, studies on the association between HDL-C levels and sarcopenia remain controversial. We therefore investigated the association between HDL-C levels and sarcopenia in elderly Koreans. **Methods**: This cross-sectional study was based on previously collected, anonymous health checkup data. Participants included 3776 individuals aged 65 years and older who underwent body composition analysis using a bioelectrical impedance meter during a health checkup in 2024. Sarcopenia was defined as an appendicular skeletal muscle index of <7.0 kg/m^2^ for males and <5.7 kg/m^2^ for females. Logistic regression analyses were performed for each variable, including HDL-C levels, to identify sarcopenia association expressed as odds ratios (ORs). Participants were further divided into four quartiles according to HDL-C levels, and comparative multivariable analyses were performed, with the quartile with the lowest HDL-C level serving as the reference. **Results**: Of the 3776 Koreans with a mean age of 70.5 years, sarcopenia was diagnosed in 23.1% (*n* = 872) of participants. Sarcopenia prevalence showed a steadily increasing trend from the lowest quartile group (Q1, *n* = 977) with HDL-C levels ≤48 mg/dL to the highest quartile group (Q4, *n* = 974) at ≥67 mg/dL (*p* < 0.001). After adjusting for sarcopenia-associated risk factors, a significant association was found between the condition and HDL-C levels (OR 1.01, 95% CI 1.00–1.02; *p* = 0.008). Q4 showed a consistent sarcopenia association compared with Q1, even after adjusting for all variables (OR 1.36, 95% CI 1.05–1.75; *p* = 0.018). **Conclusions:** In Koreans aged 65 years and older, we found an association between high HDL-C levels and sarcopenia.

## 1. Introduction

Similarly to other developed countries around the world, South Korea is aging rapidly. The country is becoming a super-aged society, where, as of December 2024, those aged 65 and over exceed 20% of the total population. Sarcopenia is a major pathological aging-associated condition, characterized not only by a decrease in skeletal muscle mass but also by a decline in muscle strength and physical function [1]. In South Korea in 2022, the prevalence of sarcopenia prevalence among adults aged 65 years and older was 6.6% for men and 9.2% for women [2].

In addition to aging, various other factors can also cause sarcopenia, including lifestyle factors such as insufficient protein intake and a lack of exercise. Anabolic resistance resulting from deficiencies in hormones such as insulin and testosterone, as well as mitochondrial dysfunction, is also cited as a major contributing factor [3]. Mitochondria are important organelles that supply energy within cells and produce reactive oxygen species as a muscle metabolism byproduct. Mitochondrial dysfunction can lead to the excessive production of reactive oxygen species, which can result in muscle damage and loss [4]. Ammonia, which increases in chronic inflammation or chronic liver disease with aging, can induce mitochondrial damage [5].

High-density lipoprotein cholesterol (HDL-C) is a commonly measured lipid profile, along with total cholesterol, triglycerides, and low-density lipoprotein cholesterol (LDL-C). HDL-C removes residual cholesterol from peripheral tissues and transports it to the liver, thereby preventing atherosclerosis and serving as a protective factor against cardiovascular disease [6]. In muscle, HDL-C contributes to energy metabolism by enhancing glucose uptake and promoting glycogen synthesis [7]. In a study comparing apolipoprotein A-I transgenic and knockout mouse models, high HDL-C maintained skeletal muscle function by lowering fasting blood glucose levels and improving glucose tolerance [8]. Furthermore, in addition to its anti-inflammatory and -oxidant effects, HDL-C has been reported to contribute to muscle maintenance by regulating mitochondrial function within muscle cells [9].

Many studies have demonstrated high HDL-C levels as a protective factor for cardiovascular disease. Considering the beneficial effects of HDL-C on muscle metabolism and function, it is hypothesized that high HDL-C levels may also act as a protective factor against sarcopenia. A meta-analysis of 16 studies, including 10 focusing on Koreans, showed a negative correlation between HDL-C levels and sarcopenia [10]. However, recent studies reported an association between high HDL-C levels and increased sarcopenia [11,12]; as such, whether this association is positive or negative remains controversial. We therefore investigated the association between HDL-C levels and sarcopenia in elderly Koreans aged 65 years and older using the appendicular skeletal muscle index (ASMI), a measure of skeletal muscle mass in the arms and legs, which is calculated by dividing the total muscle mass of the limbs by the square of a person’s height (kg/m^2^).

## 2. Materials and Methods

### 2.1. Study Participants

We targeted elderly participants aged 65 years or older who met the screening criteria for sarcopenia recommended by the Asian Sarcopenia Working Group (AWGS) [1]. Participants aged 65 to 91 years who underwent a health checkup between 1 January and 31 December 2024 were included (*n* = 3783). There were no missing data for the participants. For those who underwent health checkups more than twice during the study period (*n* = 7), only the most recent result was considered. A total of 3776 participants were finally included in the analysis. This cross-sectional study was based on retrospectively collected electronic medical records, from health checkups at a single medical institution (Samsung Changwon Hospital). Patient consent was waived because of the anonymous nature of the data. This study was approved by the Institutional Review Board of Samsung Changwon Hospital, Sungkyunkwan University (IRB No. SCMC 2025-07-013; 29 June 2025).

### 2.2. Data Collection and Variables

All participants completed a health behavior and medical history questionnaire, leaving no missing information. Smoking was defined as being a current smoker or having quit within the past five years. Only those who did not drink alcohol at all were classified as non-drinkers. Exercise was defined as ≥75 min of high-intensity exercise per week, ≥150 min of low-to-moderate-intensity exercise, or two or more sessions of strength training per week [13]. Blood samples were collected after participants had fasted for at least eight hours. Hyperlipidemia was defined as a diagnosis or a total cholesterol level of ≥240 mg/dL, a triglyceride level ≥200 mg/dL, or an LDL-C level of ≥160 mg/dL [14]. Diabetes was defined as a diagnosis or a glycated hemoglobin (HbA1c) level of ≥6.5%. Cancer was defined based on the presence or absence of a diagnosis.

### 2.3. Definition of Sarcopenia

All participants underwent body composition analysis, measured using the INBODY 970 bioelectrical impedance meter (InBody Co., Ltd., Seoul, Republic of Korea) with the subject standing upright and barefoot, according to the company’s provided specifications. Bioelectrical impedance analysis was validated and compared to MRI in assessing skeletal muscle mass [15]. Body composition analysis included height, weight, body fat percentage, visceral fat area, and skeletal muscle volume. Body mass index (BMI) was calculated using the formula weight (kg)/height (m)^2^. According to the diagnostic criteria of the World Health Organization Asia-Pacific region and the Korean Society for the Study of Obesity [16], a BMI <18.5 kg/m^2^ was classified as underweight and ≥25 kg/m^2^ was classified as obesity. Visceral obesity was defined as a cross-sectional area of visceral fat of ≥100 cm^2^. ASMI was assessed by dividing the limb muscle mass, calculated from body composition analysis, by the square of the participant’s height. Sarcopenia was diagnosed with an ASMI of <7.0 kg/m^2^ in males and <5.7 kg/m^2^ in females, according to the 2019 AWGS diagnostic criteria [1].

### 2.4. Statistical Analysis

Continuous variables were analyzed using independent *t*-tests and presented as means and standard deviations. Nominal variables were analyzed using chi-square tests and presented as frequencies and proportions relative to the total. Logistic regression analyses were performed for each variable, including HDL-C levels, to identify sarcopenia association expressed as odds ratios (ORs). Smoking, diabetes, and hyperlipidemia were included as control variables for the multivariable logistic regression analysis, as they are known risk factors for sarcopenia [17], along with other variables that showed a significant association in the univariable regression analysis. Participants were further categorized into quartiles based on their HDL-C levels, with similar numbers assigned to each quartile. ORs were compared based on the first quartile, which had the lowest HDL-C levels. In subgroup analyses, ORs for each variable were expressed using forest plots. Data were analyzed using SPSS 27.0 (IBM Co., Ltd., Armonk, NY, USA), with statistical significance defined as *p* < 0.05.

## 3. Results

### 3.1. Baseline Characteristics

The mean age of the 3776 study participants, 62.3% of which were male, was 70.5 years (Table 1). The HDL-C level criteria of participant quartiles were as follows: the first quartile (Q1) was ≤48 mg/dL (*n* = 977), the second (Q2) 49–56 mg/dL (*n* = 912), the third (Q3) 57–66 mg/dL (*n* = 913), and the fourth (Q4) ≥67 mg/dL (*n* = 974). The mean BMI was 23.9 ± 2.9 kg/m^2^, while 32.8% were obese. BMI showed a significant negative correlation with increasing HDL-C levels from Q1 (24.6 ± 2.8 kg/m^2^) to Q4 (22.8 ± 2.9 kg/m^2^). The proportion of obese individuals was higher in Q1 (42.7%) than in Q4 (21.9%) (*p* < 0.001), while the proportion of underweight subjects was higher in Q4 (5.3%) than in Q1 (1.3%) (*p* < 0.001). The prevalence of visceral obesity was higher in Q1 (30.2%) than in Q4 (22.8%) (*p* < 0.001). Approximately two-thirds (71.2%) of individuals exercised, and one-third (29.6%) engaged in strength training. The proportion of those who exercised showed a significant positive correlation, increasing from Q1 (66.1%) to Q4 (76.3%) (*p* < 0.001). The proportion of those who engaged in strength training was higher in Q4 (32.4%) than in Q1 (26.9%) (*p* = 0.030). Sarcopenia was diagnosed in 23.1% (*n* = 872) of participants, showing a steadily increasing trend from Q1 (18.8%) to Q4 (29.4%) (*p* < 0.001).

### 3.2. Association Between HDL-C Levels and Sarcopenia

**Figure 1:**
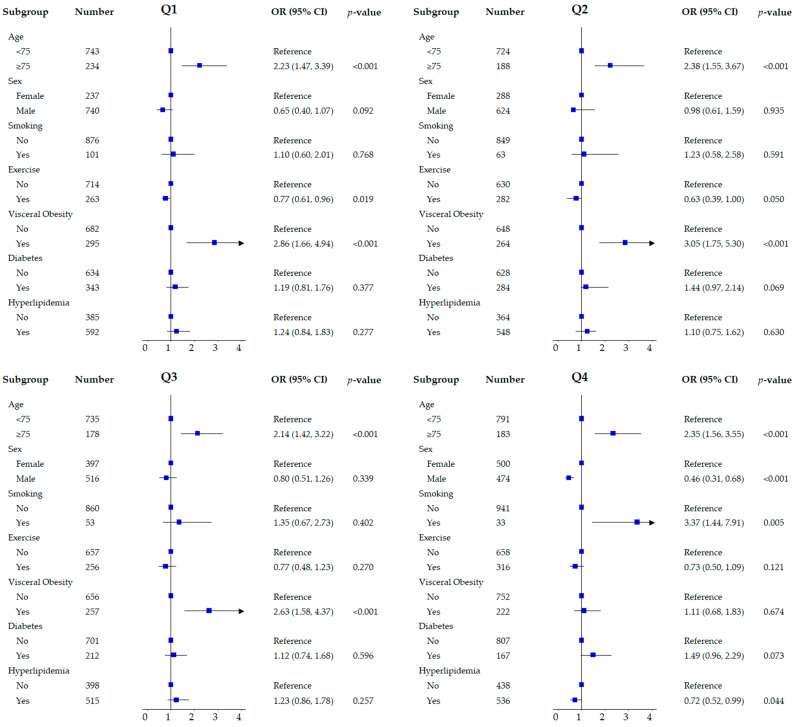
Subgroup analyses within quartiles based on HDL-C levels. OR, odds ratio; CI, confidence interval. Q1, the first quartile of HDL-C (≤48 mg/dL); Q2, the second quartile of HDL-C (49–56 mg/dL); Q3, the third quartile of HDL-C (57–66 mg/dL); Q4, the fourth quartile of HDL-C (≥67 mg/dL).

After univariable logistic regression analysis, the association between HDL-C levels and sarcopenia was significant (OR 1.02, 95% CI 1.01–1.02; *p* < 0.001) (Table 2). Age, sex, marital status, education, drinking, BMI ≥25 kg/m^2^, visceral obesity, exercise, strength training, and cancer were also significantly associated with sarcopenia. Multivariable adjustment analysis, which included recognized risk factors such as smoking, diabetes, and hyperlipidemia, revealed a consistent association between HDL-C levels and sarcopenia (OR 1.01, 95% CI 1.00–1.02; *p* = 0.008). In a comparative analysis by quartile based on Q1, with the lowest HDL-C levels, no statistical significance was observed for sarcopenia in the unadjusted model or after adjusting for demographic variables (model 2) such as age, sex, marital status, and education in Q2; however, significance was observed in Q3 and Q4 (Table 3). After adjusting for all variables (model 3), this statistical significance was lost in Q3 (OR 1.19, 95% CI 0.92–1.53; *p* = 0.188) but remained in Q4 (OR 1.36, 95% CI 1.05–1.75; *p* = 0.018). Figure 1 shows the analyses divided into subgroups within quartiles. Association with sarcopenia was significant in those aged 75 years and older, regardless of HDL-C levels. The association between visceral obesity and sarcopenia observed in other quartiles disappeared in Q4, despite the significant association observed only in this quartile for females, smokers, and those without hyperlipidemia.

## 4. Discussion

In this study, there was a negative correlation between BMI, abdominal obesity and HDL-C levels, while there was a positive correlation with exercise. Sarcopenia incidence increased as HDL-C levels increased in elderly individuals aged 65 years and older. This association was particularly evident in the highest quartile of HDL-C levels (≥67 mg/dL), even though individuals in this quartile exercised relatively more than those in the other quartiles.

Considering the known function of HDL-C in muscle cells [7,8,9], it is plausible that increasing HDL-C levels may decrease sarcopenia risk. However, recent studies on the relationship between HDL-C levels and sarcopenia have found conflicting results. In a longitudinal study of 4031 Chinese adults aged 60 years and older, the group with high HDL-C levels (≥70 mg/dL) had a significantly higher risk of developing sarcopenia and experiencing a decrease in grip strength compared to the reference group with HDL-C levels of 40–60 mg/dL [11]. A cross-sectional study including a wider age range of those 50 years and older also observed a significant association between higher HDL-C levels and increased risk of low muscle mass, as defined by computed tomography, particularly in older adult men [12]. Furthermore, HDL-C levels of >70 mg/dL consistently predicted sarcopenia in a study of 2167 U.S. adults aged 20 years and older [18].

Aging alters HDL-C metabolism and function. Insulin resistance and impaired lipolysis, which are common in elderly people with low sex-hormone levels, may impair reverse cholesterol transport through various mechanisms [19]. Furthermore, functional decline in lipid efflux and HDL-C pleiotropic effect loss may occur as a byproduct of age-dependent alterations in HDL-C particles [20]. HDL-C isolated from the elderly showed a reduced ability to promote cholesterol efflux through the ABCA1 pathway [21], as well as structural changes in apo A1 via decreased PON1 activity [22]. The dysregulation of lipid metabolism and fat accumulation within muscle cells leads to inflammation, oxidative stress, insulin resistance, and mitochondrial dysfunction, leading to sarcopenia [23]. However, the potential pathophysiological mechanisms linking age-related changes in HDL-C metabolism and function to sarcopenia are not completely understood. Further studies, including in vivo, are needed to substantiate these mechanisms.

In addition, we might consider molecular mechanisms that change with HDL-C levels. In terms of cardiovascular protection, endothelial progenitor cells (EPCs) contribute to angiogenesis [24]. In an experimental in vivo study, HDL-C increased the angiogenic capacity of EPC and activated the phosphatidylinositol-3 kinase signaling pathway at low concentrations (0.5–5 mg/dL), promoting angiogenesis. However, at medium to high concentrations (40–80 mg/dL), HDL-C promotes EPC senescence and activates the rho-associated kinase signaling pathway, inhibiting angiogenesis [25]. This inhibition can lead to muscle loss due to insufficient oxygen and nutrient supply, which may be considered as a mechanism that supports the positive correlation between high HDL-C levels and sarcopenia.

Changes in HDL-C function during chronic inflammation can be related to its impact on sarcopenia. During chronic inflammation, tissue changes due to local inflammatory responses and immune cell infiltration lead to impaired muscle protein synthesis and breakdown [26]. As sarcopenia stages progress, the levels of various inflammatory biomarkers appear to be higher, which may be an adjuvant tool in diagnosing and classifying the severity of sarcopenia [27]. While HDL-C generally possesses anti-inflammatory properties, it can lose its protective function or even become pro-inflammatory in the context of aging and chronic diseases [28]. In a study of individuals with coronary artery disease and very high HDL-C levels (≥84 mg/dL), its pro-inflammatory properties were significantly higher compared to in healthy controls [29]. A study of 25,541 participants also demonstrated that extremely high HDL-C levels (≥100 mg/dL) were associated with a greater risk of mortality and higher levels of inflammatory markers [30]. Although the exact mechanism remains unknown, the significant association between high HDL-C levels and sarcopenia observed in our study suggests that further researching the functional and inflammatory aspects of HDL-C in elderly populations is crucial.

This study has several limitations that should be considered. Because our definition of sarcopenia did not include functional aspects such as grip strength, it may not properly reflect sarcopenic obesity, which is characterized by sufficient muscle mass but reduced strength. The cross-sectional study design precluded establishing causal relationships between variables and outcomes. Furthermore, it was impossible to assess variability and biological changes in HDL-C measurements over time, which may act as a residual risk factor for various sarcopenia-associated health outcomes [31,32]. Questionnaire-based information on disease status and lifestyle habits could not completely rule out the possibility of recall bias. The institutional health screening guidelines recommended at least 8 h of fasting; however, 12 h of fasting was not recommended, which could potentially bias our study. Finally, this study was conducted at a single medical center and focused only on Koreans, limiting its generalizability. Nonetheless, the strength of this study was that high HDL-C levels may be associated with sarcopenia in elderly individuals, particularly non-obese women, aged 75 years or older with very high HDL-C levels (≥67 mg/dL). This finding offers clinical implications for careful sarcopenia monitoring in this population.

## 5. Conclusions

We found a positive association between HDL-C levels and sarcopenia in Koreans aged 65 years and older, particularly in participants with HDL-C levels ≥67 mg/dL. Although this association was not clearly evident across all participants, it should be taken into consideration when treating elderly individuals with exceptionally high HDL-C levels. Future research clarifying the pathophysiological mechanisms of HDL-C and sarcopenia will allow us to identify causal relationships and understand their implications in real-world clinical settings.

## Figures and Tables

**Table 1:** Baseline characteristics of study participants.

Characteristics	Overall (*n* = 3776)	Q1 (*n* = 977)	Q2 (*n* = 912)	Q3 (*n* = 913)	Q4 (*n* = 974)	*p*-Value
Age (year)	70.5 ± 5.0	71.2 ± 5.5	70.3 ± 4.9	70.4 ± 4.7	70.2 ± 4.7	<0.001
Sex						
Male	2354 (62.3)	740 (75.7)	624 (68.4)	516 (56.5)	474 (48.7)	<0.001
Female	1422 (37.7)	237 (24.3)	288 (31.6)	397 (43.5)	500 (51.3)	<0.001
Height (cm)	162.4 ± 8.6	164.3 ± 8.3	163.4 ± 8.5	161.4 ± 8.6	160.6 ± 8.4	<0.001
Weight (kg)	63.1 ± 10.2	66.6 ± 10.2	64.6 ± 10.1	62.2 ± 9.4	59.1 ± 9.4	<0.001
BMI (kg/m^2^)	23.9 ± 2.9	24.6 ± 2.8	24.1 ± 2.8	23.8 ± 2.8	22.8 ± 2.9	<0.001
<18.5	88 (2.3)	13 (1.3)	11 (1.2)	12 (1.3)	52 (5.3)	<0.001
18.5–24.9	2449 (64.9)	547 (56.0)	571 (62.6)	622 (68.1)	709 (72.8)	<0.001
≥25	1239 (32.8)	417 (42.7)	330 (36.2)	279 (30.6)	213 (21.9)	<0.001
Visceral obesity	1038 (27.5)	295 (30.2)	264 (28.9)	257 (28.1)	222 (22.8)	<0.001
Married	2873 (76.1)	760 (77.8)	710 (77.9)	681 (74.6)	722 (74.1)	0.098
Educated ^1^	1332 (35.3)	335 (34.3)	341 (37.4)	333 (36.5)	323 (33.2)	0.197
Smoking	250 (6.6)	101 (10.3)	63 (6.9)	53 (5.8)	33 (3.4)	<0.001
Drinking	1958 (51.9)	494 (50.6)	487 (53.4)	468 (51.3)	509 (52.3)	0.633
Exercise	2688 (71.2)	646 (66.1)	648 (71.1)	651 (71.3)	743 (76.3)	<0.001
Strength training	1117 (29.6)	263 (26.9)	282 (30.9)	256 (28.0)	316 (32.4)	0.030
HbA1c (%)	6.0 ± 0.8	6.2 ± 0.9	6.1 ± 0.9	5.9 ± 0.7	5.8 ± 0.6	<0.001
Diabetes	1006 (26.6)	343 (35.1)	284 (31.1)	212 (23.2)	167 (17.1)	<0.001
Cancer	427 (11.3)	114 (11.7)	86 (9.4)	111 (12.2)	116 (11.9)	0.227
Hyperlipidemia	2191 (58.0)	592 (60.6)	548 (60.1)	515 (56.4)	536 (55.0)	0.032
hs-CRP	1.0 ± 1.2	1.2 ± 1.4	1.0 ± 1.3	0.9 ± 1.2	0.7 ± 0.9	<0.001
Total cholesterol	172.8 ± 40.6	158.0 ± 41.0	169.1 ± 39.8	174.6 ± 38.4	189.3 ± 36.6	<0.001
Triglyceride	106.5 ± 57.8	139.7 ± 74.0	108.4 ± 51.6	96.7 ± 43.4	80.5 ± 36.8	<0.001
LDL-C	103.6 ± 37.7	97.8 ± 37.6	104.7 ± 38.1	104.7 ± 38.0	107.4 ± 36.2	<0.001
HDL-C	58.3 ± 14.3	42.0 ± 5.0	52.7 ± 2.2	61.2 ± 2.8	77.2 ± 9.7	<0.001
ASMI	7.0 ± 1.0	7.3 ± 1.0	7.1 ± 1.0	6.9 ± 1.0	6.7 ± 1.0	<0.001
Sarcopenia	872 (23.1)	184 (18.8)	188 (20.6)	214 (23.4)	286 (29.4)	<0.001

Values are presented as number (%) or mean ± standard deviation. Q1, the first quartile of HDL-C (≤48 mg/dL); Q2, the second quartile of HDL-C (49–56 mg/dL); Q3, the third quartile of HDL-C (57–66 mg/dL); Q4, the fourth quartile of HDL-C (≥67 mg/dL). BMI, body mass index; HbA1c, glycated hemoglobin; hs-CRP, high-sensitivity C-reactive protein; LDL-C, low-density lipoprotein cholesterol; HDL-C, high-density lipoprotein cholesterol; ASMI, appendicular skeletal mass index. ^1^ University or higher degree.

**Table 2:** Logistic regression analyses to identify association for sarcopenia.

Variables	Univariable Adjustment		Multivariable Adjustment ^1^	
	OR (95% CI)	*p*-Value	OR (95% CI)	*p*-Value
Age	1.08 (1.07–1.10)	<0.001	1.09 (1.07–1.11)	<0.001
Sex	0.55 (0.47–0.64)	<0.001	0.63 (0.50–0.78)	<0.001
Marital status	0.73 (0.61–0.86)	<0.001	0.99 (0.80–1.23)	0.946
Education	0.96 (0.92–1.00)	0.022	0.96 (0.92–1.00)	0.066
Smoking	1.08 (0.80–1.46)	0.612	1.54 (1.09–2.19)	0.014
Drinking	0.64 (0.55–0.74)	<0.001	1.01 (0.84–1.23)	0.885
Obesity ^2^	0.08 (0.06–0.10)	<0.001	0.05 (0.37–0.69)	<0.001
Visceral obesity	0.72 (0.60–0.86)	<0.001	2.06 (1.60–2.66)	<0.001
Exercise	0.67 (0.57–0.79)	<0.001	0.80 (0.65–0.99)	0.038
Strength training	0.63 (0.53–0.75)	<0.001	0.78 (0.62–0.97)	0.027
Diabetes	1.11 (0.94–1.31)	0.232	1.31 (1.07–1.60)	0.008
Cancer	1.26 (1.00–1.59)	0.046	1.07 (0.82–1.39)	0.618
Hyperlipidemia	0.88 (0.76–1.03)	0.101	1.06 (0.89–1.27)	0.512
hs-CRP	1.01 (0.95–1.08)	0.702		
Triglyceride	1.00 (1.00–1.00)	0.110		
LDL-C	1.00 (1.00–1.00)	0.075		
HDL-C	1.02 (1.01–1.02)	<0.001	1.01 (1.00–1.02)	0.008

OR, odds ratio; CI, confidence interval; hs-CRP, high-sensitivity C-reactive protein; LDL-C, low-density lipoprotein cholesterol; HDL-C, high-density lipoprotein cholesterol. ^1^ Adjusted for age, sex, marital status, education, smoking, drinking, obesity, visceral obesity, exercise, strength training, diabetes, cancer, and hyperlipidemia. ^2^ Body mass index ≥25 kg/m^2^.

**Table 3:** Association between HDL-C levels and sarcopenia categorized into quartiles.

Quartiles	OR (95% CI)					
	Model 1	*p*-Value	Model 2	*p*-Value	Model 3	*p*-Value
Q1	Reference		Reference		Reference	
Q2	1.12 (0.89–1.40)	0.331	1.19 (0.94–1.50)	0.150	1.15 (0.89–1.49)	0.294
Q3	1.32 (1.06–1.65)	0.014	1.31 (1.04–1.65)	0.020	1.19 (0.92–1.53)	0.188
Q4	1.79 (1.45–2.21)	<0.001	1.77 (1.41–2.21)	<0.001	1.36 (1.05–1.75)	0.018

OR, odds ratio; CI, confidence interval. Q1, the first quartile of HDL-C (≤48 mg/dL); Q2, the second quartile of HDL-C (49–56 mg/dL); Q3, the third quartile of HDL-C (57–66 mg/dL); Q4, the fourth quartile of HDL-C (≥67 mg/dL). Model 1, unadjusted. Model 2, adjusted for age, sex, marital status, and education. Model 3, adjusted for age, sex, marital status, education, smoking, drinking, obesity, visceral obesity, exercise, strength training, diabetes, cancer, and hyperlipidemia.

## Data Availability

The data presented in this study are available on request from the corresponding author. (This data was collected from individuals who underwent health checkups at Samsung Changwon Hospital and is therefore not publicly available.)

## Abbreviations

The following abbreviations are used in this manuscript:

HDL-C	High-density lipoprotein cholesterol
LDL-C	Low-density lipoprotein cholesterol
ASMI	Appendicular skeletal muscle index
AWGS	Asian Working Group for Sarcopenia
HbA1c	glycated hemoglobin
BMI	Body mass index
ORs	Odds ratios
CI	Confidence interval
EPC	Endothelial progenitor cells
